# Automated Bone Scan Index as a quantitative imaging biomarker in metastatic castration-resistant prostate cancer patients being treated with enzalutamide

**DOI:** 10.1186/s13550-016-0173-z

**Published:** 2016-03-09

**Authors:** Aseem Anand, Michael J. Morris, Steven M. Larson, David Minarik, Andreas Josefsson, John T. Helgstrand, Peter S. Oturai, Lars Edenbrandt, Martin Andreas Røder, Anders Bjartell

**Affiliations:** Department of Translational Medicine, Division of Urological Cancers, Lund University, Waldenströms Gata 5, Malmö, SE 205 02 Sweden; Department of Medicine, Memorial Sloan Kettering Cancer Center, New York, USA; Weil Cornell Medical College, New York, USA; Department of Radiology, Memorial Sloan Kettering Cancer Center, New York, USA; Department of Radiation Physics, Skåne University Hospital, Lund University, Malmö, Sweden; Department of Urology, Institute of Clinical Sciences, Sahlgrenska Academy, Gothenburg, Sweden; Copenhagen Prostate Cancer Center, Department of Urology, Rigshospitalet, University of Copenhagen, Copenhagen, Denmark; Department of Clinical Physiology, Nuclear Medicine and PET, Copenhagen University Hospital, Copenhagen, Denmark; Department of Nuclear Medicine, Skåne University Hospital, Malmö, Sweden; Department of Urology, Skåne University Hospital, Malmö, Sweden

**Keywords:** Bone Scan Index (BSI), Imaging biomarker, Bone scan, Enzalutamide, Metastatic castration-resistant prostate cancer (mCRPC)

## Abstract

**Background:**

Having performed analytical validation studies, we are now assessing the clinical utility of the upgraded automated Bone Scan Index (BSI) in metastatic castration-resistant prostate cancer (mCRPC). In the present study, we retrospectively evaluated the discriminatory strength of the automated BSI in predicting overall survival (OS) in mCRPC patients being treated with enzalutamide.

**Methods:**

Retrospectively, we included patients who received enzalutamide as a clinically approved therapy for mCRPC and had undergone bone scan prior to starting therapy. Automated BSI, prostate-specific antigen (PSA), hemoglobin (HgB), and alkaline phosphatase (ALP) were obtained at baseline. Change in automated BSI and PSA were obtained from patients who have had bone scan at week 12 of treatment follow-up. Automated BSI was obtained using the analytically validated EXINI Bone^BSI^ version 2. Kendall’s tau (*τ*) was used to assess the correlation of BSI with other blood-based biomarkers. Concordance index (C-index) was used to evaluate the discriminating strength of automated BSI in predicting OS.

**Results:**

Eighty mCRPC patients with baseline bone scans were included in the study. There was a weak correlation of automated BSI with PSA (*τ* = 0.30), with HgB (*τ* = −0.17), and with ALP (*τ* = 0.56). At baseline, the automated BSI was observed to be predictive of OS (C-index 0.72, standard error (SE) 0.03). Adding automated BSI to the blood-based model significantly improved the C-index from 0.67 to 0.72, *p* = 0.017. Treatment follow-up bone scans were available from 62 patients. Both change in BSI and percent change in PSA were predictive of OS. However, the combined predictive model of percent PSA change and change in automated BSI (C-index 0.77) was significantly higher than that of percent PSA change alone (C-index 0.73), *p* = 0.041.

**Conclusions:**

The upgraded and analytically validated automated BSI was found to be a strong predictor of OS in mCRPC patients. Additionally, the change in automated BSI demonstrated an additive clinical value to the change in PSA in mCRPC patients being treated with enzalutamide.

## Background

Bone metastasis is present in 90 % of patients with metastatic castration-resistant prostate cancer (mCRPC) [1]. In such patients, the bone scan is the standard imaging modality to assess change in skeletal disease burden. However, the interpretation of the bone scan has significant limitations. The qualitative manual assessment of the bone scan is dependent on the skill and expertise of the local reader. Additionally, the criteria of counting new lesions to identify disease progression do not account for changes in disease distribution, increase in disease burden of existing lesions, or increase in confluent disease. Therefore, there is a need to qualify a fully quantitative assessment of disease burden in bone to detect post-treatment changes that are clinically relevant.

The manual Bone Scan Index (BSI), developed at the Memorial Sloan Kettering Cancer Center, is a fully quantitative assessment of bone scans that accounts for the metastatic lesions, as the percentage of total skeletal mass [2]. The manual BSI and its changes at treatment follow-up have been shown to have a significant association with overall survival (OS) [3, 4]. Despite showing its clinical utility, the manual BSI has not been adopted in routine clinical practice due to the laborious process of manual calculations.

To overcome the limitations of manual assessment, the BSI methodology was automated using a computerized image analysis system that employed an artificial neural network [5]. With the computer automation, the time of detecting metastatic lesion and calculating the BSI was reduced from an average 20 min (by an experienced reader) to 5 s per patient.

Recently, in a multi-institutional effort, we performed the analytical validation of an upgraded automated BSI platform in assessing change in bone scan of metastatic prostate cancer [6]. The consistent linearity of the upgraded BSI platform overcame the limitation of its predecessor in underestimating the BSI values in patients with a higher (>5 BSI) tumor burden [7]. Additionally, the upgraded automated BSI demonstrated that with minimal manual supervision, it can standardize the inter-operator variability in the assessment of change in bone scan. The result demonstrated the reliability of automated BSI in assessing change in bone scan and served as the foundation for future clinical studies.

Treatment with enzalutamide has shown to improve progression free survival and prolong OS in patients with mCRPC [8, 9]. In this hypothesis-generating retrospective study, we assessed the discriminatory strength of the upgraded automated BSI in predicting OS in mCRPC patients being treated with enzalutamide. As a part of the clinical qualification effort, the study here is aimed to provide justification for prospective validation of the upgraded and analytically validated automated BSI as a first quantitative imaging biomarker that can add clinical value to the treatment management of the mCRPC patients.

## Methods

### Study design

The retrospective study would aim to generate the hypothesis, for prospective validation, that as an imaging biomarker the automated BSI values provide distinct information and have an additive clinical value over the available blood-based markers. To evaluate the automated BSI as a marker with distinct information, the values of the BSI were associated with the values of the blood-based markers. To evaluate the additive clinical value of automated BSI, discriminatory strength of the blood-based biomarkers was assessed with and without the addition of automated BSI values in predicting OS of mCRPC patients being treated with enzalutamide.

### Patients

All mCRPC patients who initiated treatment with Food and Drug Administration (FDA)-approved enzalutamide, from December 2012 to December 2014, at Skåne University Hospital in Malmö, Sweden, and at Copenhagen University Hospital (Rigshospitalet) in Copenhagen, Denmark, were considered for the study. CRPC was defined as the patients who had failed androgen deprivation therapy and had been clinically documented to have prostate-specific antigen (PSA) and/or radiological progression despite castrate level of testosterone (<20 ng/dL). Prior radiological scans were used to confirm the presence of metastasis. The mCRPC patients, who had undergone bone scan, as part of the clinical routine, before initiating enzalutamide treatment, were enrolled in the retrospective analysis. In these patients, we retrospectively collected the PSA, hemoglobin (HgB), and alkaline phosphatase (ALP) prior to initiating treatment with enzalutamide. Change in automated BSI and percent change in PSA were obtained from patients who had bone scan available at 12 week (±4 weeks) of treatment follow-up.

The retrospective study was performed in accordance with the Declaration of Helsinki. The ethical permission for the retrospective study and individual patient consent was obtained at the regional ethical review board at Lund University, Sweden, and at Rigshospitalet, Denmark.

### Bone scan

The whole-body bone scan was obtained after 3 h of a single intravenous injection of 600 MBq Tc-99m methylene diphosphonate. Whole-body images with anterior and posterior views (scan speed 10 cm/min, 256 × 1024 matrix) were obtained using a gamma camera equipped with low-energy, high-resolution parallel hole collimators (Maxxus; General Electric, Milwaukee, WI, USA (Malmö); Precedence; Philips Healthcare, Eindhoven, The Netherlands (Copenhagen)). Energy discrimination was provided by a 15 % window centered on the 140 keV of Tc-99m.

### Automated BSI analysis

The upgraded EXINI Bone^BSI^ software (version 2), developed by EXINI Diagnostics AB (Lund, Sweden), was used to analyze the retrospectively collected bone scans and to generate automated BSI. The methodology of the automated platform has been described in detail in a previous study [7]. In summary, the different anatomical regions of the skeleton are segmented followed by detection and classification of the abnormal hotspots as metastatic lesions. The weight fraction of the skeleton for each metastatic hotspot is calculated, and the BSI is calculated as the sum of all such fractions.

### Statistical analysis

As a hypothesis generating retrospective study, no prior assumptions were made for the clinical utility of automated BSI to render power calculations. To evaluate the automated BSI as a distinct and independent variable, we used the Kendall’s tau (*τ*) and the Spearman’s rank correlation coefficient to determine the association between automated BSI and other blood-based biomarkers. Kendall’s tau and the Spearman’s rank correlation are the two available non-parametric rank correlations that measure the strength of association between two separate variables.

Kaplan Meier method was used to estimate the median OS of the baseline and of the treatment follow-up patient cohorts. Concordance index (C-index) and its standard error (SE) was used to evaluate the discriminatory strength of the automated BSI in predicting OS [10]. To determine the additive strength of automated BSI as a biomarker, we compared the discrimination, C-index values [11], of the blood-based model to that of the blood-based model incorporating the automated BSI. C-index would allow the evaluation of the additive clinical value of automated BSI as a continuous covariate over the blood biomarkers, without stratifying the data with an arbitrary threshold. A strong concordance would indicate that the covariate is highly informative in predicting the relative risk of death between any two patients at a given time. Statistical significance for each statistical test was set at 0.05 (two-tailed). All statistical analyses were performed using R software version 3.1.2.

## Results

### Patients

**Fig. 1:**
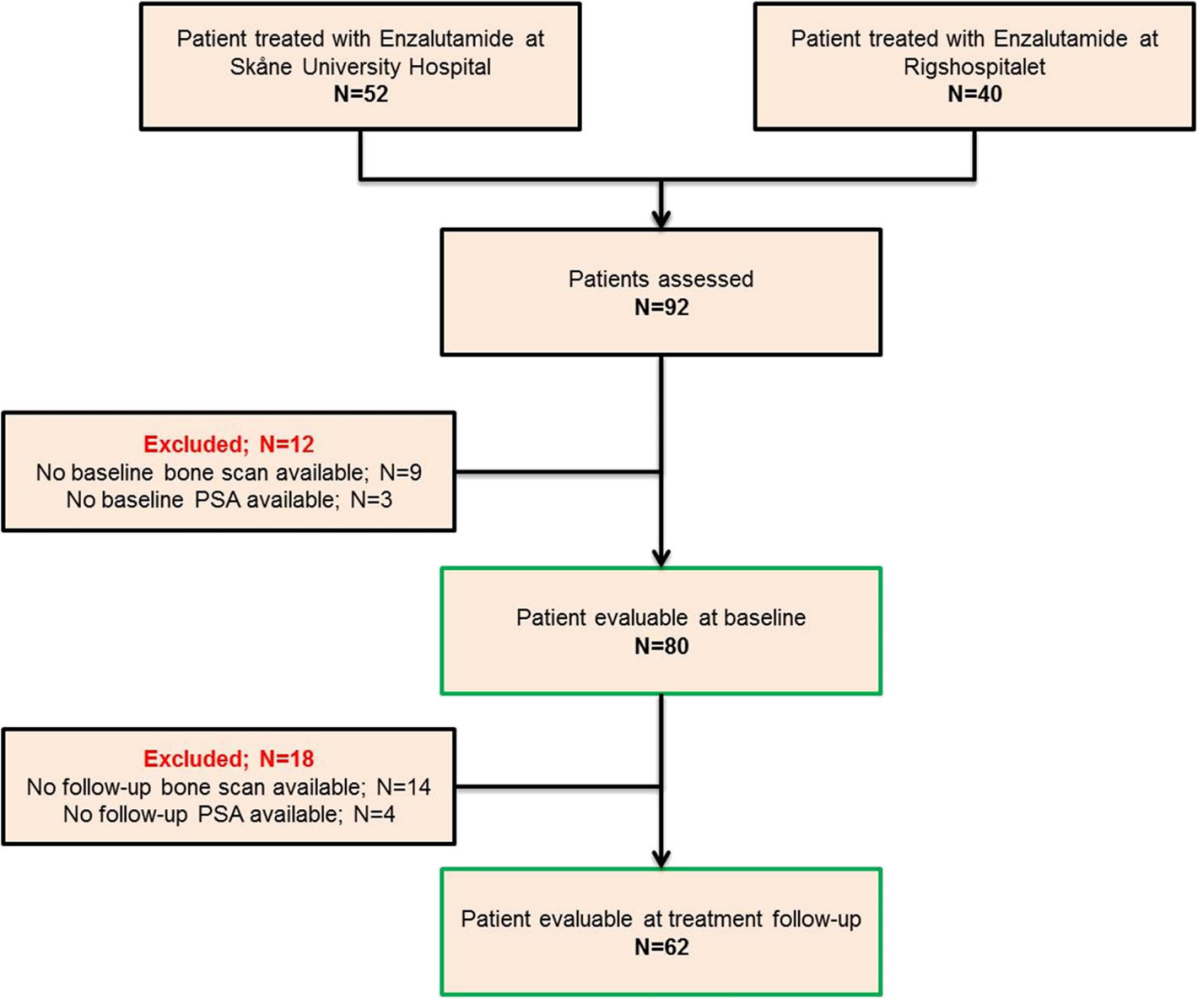
Flow chart of evaluable patients available for analyses

Eighty of the eligible mCRPC patients had their baseline bone scans available for automated BSI analysis with median follow-up time of 56 weeks. Sixty-two of the eighty mCRPC patients had available bone scans at week 12 of treatment follow-up. Figure 1 illustrates the evaluable patients available at baseline and at treatment follow-up. The demographics, prior treatment history and baseline characteristics of the evaluable mCRPC patients are summarized in Table 1.

**Table 1:** Demographic and clinical history of patients that qualified for the survival analysis

Demographics	Patients evaluable at baseline	Patients evaluable at 12-week treatment follow-up
	(*N* = 80)	(*N* = 62)
	Median (range)	
Age	71 (54–84)	71 (54–84)
PSA at diagnosis	46 (3.7–4625)	46 (3.7–1018)
Primary treatment	*N* (%)	
Radiation therapy	22 (28)	19 (30)
Radical prostatectomy	13 (16)	9 (15)
Prior systemic treatment		
Prior ADT	80 (100)	62 (100)
Prior chemo	64 (80)	47 (75)
Covariates	Median (range)	
PSA (ng/mL)	157.5 (1.1–5460)	149 (1.1–5460)
ALP (U/L)	124.3 (41.2–1058)	–
HgB (mmol/L)	7.5 (5.2–10.0)	–
BSI	2.7 (0.01–21.11)	2.6 (0.01–21.11)
	*N* (%)	
Deaths	56 (70)	40 (65)

### Baseline analysis

The Kendall’s tau correlations and Spearman correlation of the automated BSI with the blood-based biomarkers are detailed in Table 2. There was a weak correlation observed between automated BSI and all the available blood-based biomarkers (*N* = 80). With 56 (70 %) events, the median survival time for the 80 patients was 59 weeks (95 % confidence interval, 34–84 weeks). The C-index analysis at baseline of the 80 mCRPC patients are summarized in Table 3 (A). The automated BSI at baseline was observed to be predictive of OS (C-index 0.72). The addition of the automated BSI to the blood-based model that included PSA, ALP, and HgB significantly improved the C-index increased from 0.67 to 0.72, *p* = 0.017.

**Table 2:** Non-parametric Kendall’s tau and Spearman correlation of automated BSI against blood-based biomarkers (*N* = 80)

Blood-based biomarkers	Kendall’s tau (against BSI)	*p* value	Spearman (against BSI)	*p* value
PSA	0.30	0.00015	0.41	0.00011
ALP	0.56	<0.0001	0.64	<0.0001
HgB	−0.17	0.0272	−0.25	0.0265

**Table 3:** A: C-index analysis of with and without the addition of BSI to blood based biomarkers at baseline. B: C-index analysis of change in BSI and PSA at treatment follow-up

	C-index	Confidence interval	SE
A. Baseline analysis^a^ (dead/total = 56/80)			
BSI	0.71	0.64–0.77	0.03
PSA	0.65	0.57–0.72	0.03
ALP	0.67	0.58–0.75	0.03
HgB	0.26	0.20–0.32	0.03
PSA + ALP + HgB	0.67	0.58–0.74	0.03
PSA + ALP + HgB + BSI	0.72	0.64–0.78	0.03
B. Change (∆) at week 12 (dead/total = 40/62)			
∆ BSI	0.75	0.68–0.81	0.05
∆ PSA	0.73	0.66–0.78	0.05
∆ PSA + ∆ BSI	0.77	0.71–0.82	0.05

^a^BSI, PSA, ALP, and HgB values were observed skewed and therefore logarithmically transformed

### Treatment follow-up analysis

**Fig. 2:**
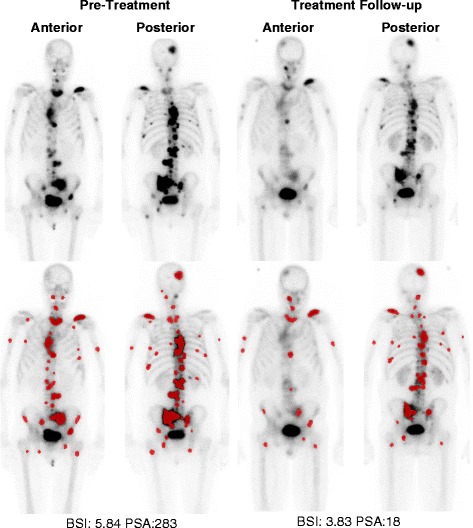
Illustrative example of BSI analysis and PSA change after treatment with enzalutamide at 12-week treatment follow-up. Lesions detected and classified as metastatic by the neural network of the automated EXINI platform for BSI calculation are highlighted in *red*

Change in the automated BSI (median = 0.05, interquartile range = [−] 0.28–1.43) and the percent change in PSA (median = [−] 0.60, interquartile range = [−] 0.86–[−] 0.12) at treatment follow-up was calculated in all 62 patients. An example of the BSI and PSA change at treatment follow-up is shown in Fig. 2. The median survival time for 62 patients, with treatment follow-up data, was 83 weeks (95 % confidence interval, 59–163 weeks). The C-index analysis of on-treatment change in BSI and PSA is shown in Table 3 (B). The combined predictive model of percent PSA change and change in automated BSI (C-index 0.77) was observed to be significantly higher than that of percent PSA change alone (C-index 0.73), *p* = 0.041.

## Discussion

Bone scan remains the standard imaging modality to assess radiographic progression-free survival in patients with mCRPC. As a primary endpoint, the progression-free survival has been accepted by FDA to show the treatment efficacy of novel therapy in patients with mCRPC [12]. There is an unmet need for a quantitative and a reproducible assessment of bone scan that generate reliable data in multi-institutional registration trials. The study presented here is an incremental progress of the automated BSI towards its qualification as an imaging biomarker with clinical relevance in mCRPC patients. Compared to its predecessor, which underestimated the BSI values in patients with a high tumor burden (>5 BSI), the upgraded EXINI Bone^BSI^ (version 2) platform generates a consistent BSI assessment for patient with low and high tumor burdens. The performance of the commercially available platform was recently evaluated in a multi-institutional analytical validation study [6]. In this hypothesis-generating study, we have demonstrated that the upgraded automated BSI is a strong predictor of OS with an additive clinical value to the existing blood-based biomarkers.

In baseline survival analysis, our study demonstrated that the automated BSI was predictive of OS (C-index 0.72). The independent and additive clinical value of automated BSI was demonstrated in the significant improvement of the blood-based model’s discrimination in predicting OS (C-index from 0.67 to 0.72). Additionally, the weak correlation between the automated BSI and the blood-based biomarkers implied that the automated BSI is an independent marker with distinct quantitative information that can add to the clinical evaluation of patients with skeletal metastasis. Our data supports the initial findings of the original manual BSI studies that have demonstrated significant and strong association with OS in patients with mCRPC [3]. The data is also in agreement with the previous version of a computer-automated BSI platform that demonstrated the prognostic value of the automated BSI in newly diagnosed and in mCRPC patients [7, 13]. The result should encourage investigators to evaluate the role of automated BSI in existing prognostic models for metastatic prostate cancer, which noticeably lacked a quantitative imaging parameter indicative of skeletal disease burden [1, 14]. The baseline analysis in our study was limited by the unavailability of lactate dehydrogenase and albumin, both of which have demonstrated an association with OS and are part of the baseline prognostic model in mCRPC patients.

Enzalutamide prevents the translocation of the androgen receptor to nucleus that prevents the activation of some proliferative genes, which includes PSA. In its registration studies, enzalutamide has shown improvement in OS with early measure of response shown by percent PSA decline at week 12 [8, 15]. In agreement to these studies, we observed a marked decrease in percent PSA change at week 12 of treatment with enzalutamide (median = [−] 0.60, IQR = [−] 0.12–[−] 0.86). The study also demonstrated that the combined model, including the change in automated BSI and the percent change in PSA, was significantly stronger in discriminating for OS than the percent change in PSA alone. The data is unique in its attempt to establish that with consistent linearity, the change in automated BSI of the upgraded platform is not only reproducible but is also clinically relevant. In Japan, Mitsui et al. has similarly demonstrated improvement in C-index with addition of automated BSI to PSA (from 0.62 to 0.66) [16]. However, it should be noted that the platform used in the Mitsui et al. work, BONENAVI®, has been uniquely trained on a data set consisting exclusively of Japanese patients and therefore caters to a distinct population base [17].

As an indirect measurement of disease burden, PSA and bone scan lesions are known to increase temporarily within few weeks of response to an effective therapy. This increase, specifically observed in bone scan, is commonly referred to as flare phenomenon. Comprehensive investigations in patients with metastatic prostate cancer have shown the flare to peak at weeks 6–8 of treatment follow-up [18, 19]. Therefore, Prostate Cancer Working Group 2 (PCWG2) criteria recommend that early changes (before 12 weeks) in PSA and radionuclide bone scan should be ignored [20]. Although the evidence of the flare phenomenon in mCRPC patients being treated with enzalutamide is quite limited, if not non-existent, the current study evaluated the change in automated BSI and in PSA at week 12 of treatment follow-up. Following the PCWG2 recommendation, the analysis at week 12 of treatment follow-up minimizes but does not overcome the risk of flare and its limitation on the clinical evaluation of automated BSI.

The current study was limited in its scope as a retrospective analysis. However, with its enrollment criteria, the study was controlled for the standardized time point analysis at pre-treatment and at 12-week treatment follow-up for each of the respective covariates. A reasonable large number of patients were included from the two separate hospitals to avoid site selection biases. The data presented here, using an upgraded and analytically validated platform, confirms the original clinical findings of the manual BSI studies. More importantly, the study is part of the continual effort to clinically validate automated BSI as an imaging biomarker to quantify the change in total skeletal tumor burden that is clinically relevant. The computer-automated BSI represents an opportunity to realize the clinical potential of a standard imaging modality, bone scan, which has been limited by the variability of visual assessment.

## Conclusions

In the present study, our data has demonstrated that the analytically validated automated BSI is an independent and a strong predictor of OS in mCRPC patients. The study also found that the change in automated BSI has an additive clinical value to the change in PSA in mCRPC patients being treated with enzalutamide. The data presented here confirms the initial findings of the original manual BSI studies and serve as the foundation for future prospective studies aimed to clinically validate automated BSI as an imaging biomarker in mCRPC patients.

## References

[1] Halabi S, Lin CY, Kelly WK, Fizazi KS, Moul JW, Kaplan EB, Morris MJ, Small EJ (2014). Updated prognostic model for predicting overall survival in first-line chemotherapy for patients with metastatic castration-resistant prostate cancer. J Clin Oncol.

[2] Imbriaco M, Larson SM, Yeung HW, Mawlawi OR, Erdi Y, Venkatraman ES, Scher HI (1998). A new parameter for measuring metastatic bone involvement by prostate cancer: the Bone Scan Index. Clin Cancer Res.

[3] Sabbatini P, Larson SM, Kremer A, Zhang ZF, Sun M, Yeung H, Imbriaco M, Horak I, Conolly M, Ding C, Ouyang P, Kelly WK, Scher HI (1999). Prognostic significance of extent of disease in bone in patients with androgen-independent prostate cancer. J Clin Oncol.

[4] Dennis ER, Jia X, Mezheritskiy IS, Stephenson RD, Schoder H, Fox JJ, Heller G, Scher HI, Larson SM, Morris MJ (2012). Bone scan index: a quantitative treatment response biomarker for castrationresistant metastatic prostate cancer. J Clin Oncol.

[5] Sadik M, Suurkula M, Hoglund P, Jarund A, Edenbrandt L (2009). Improved classifications of planar whole-body bone scans using a computer-assisted diagnosis system: a multicenter, multiple-reader, multiple-case study. J Nucl Med.

[6] Anand A, Morris MJ, Kaboteh R, Bath L, Sadik M, Gjertsson P, Lomsky M, Edenbrandt L, Minarik D, Bjartell A (2016). Analytic Validation of the Automated Bone Scan Index as an Imaging Biomarker to Standardize Quantitative Changes in Bone Scans of Patients with Metastatic Prostate Cancer. J Nucl Med.

[7] Ulmert D, Kaboteh R, Fox JJ, Savage C, Evans MJ, Lilja H, Abrahamsson PA, Bjork T, Gerdtsson A, Bjartell A, Gjertsson P, Hoglund P, Lomsky M, Ohlsson M, Richter J, Sadik M, Morris MJ, Scher HI, Sjostrand K, Yu A, Suurkula M, Edenbrandt L, Larson SM (2012). A novel automated platform for quantifying the extent of skeletal tumour involvement in prostate cancer patients using the Bone Scan Index. Eur Urol.

[8] Scher HI, Fizazi K, Saad F, Taplin ME, Sternberg CN, Miller K, de Wit R, Mulders P, Chi KN, Shore ND, Armstrong AJ, Flaig TW, Flechon A, Mainwaring P, Fleming M, Hainsworth JD, Hirmand M, Selby B, Seely L, de Bono JS, Investigators, Affirm (2012). Increased survival with enzalutamide in prostate cancer after chemotherapy. N Engl J Med.

[9] Beer TM, Tombal B (2014). Enzalutamide in metastatic prostate cancer before chemotherapy. N Engl J Med.

[10] Pencina MJ, D'Agostino RB (2004). Overall C as a measure of discrimination in survival analysis: model specific population value and confidence interval estimation. Stat Med.

[11] Haibe-Kains B, Desmedt C, Sotiriou C, Bontempi G (2008). A comparative study of survival models for breast cancer prognostication based on microarray data: does a single gene beat them all?. Bioinformatics.

[12] Kluetz PG, Ning YM, Maher VE, Zhang L, Tang S, Ghosh D, Aziz R, Palmby T, Pfuma E, Zirkelbach JF, Mehrotra N, Tilley A, Sridhara R, Ibrahim A, Justice R, Pazdur R (2013). Abiraterone acetate in combination with prednisone for the treatment of patients with metastatic castration-resistant prostate cancer: U.S. Food and Drug Administration drug approval summary. Clin Cancer Res.

[13] Armstrong AJ, Kaboteh R, Carducci MA, Damber JE, Stadler WM, Hansen M, Edenbrandt L, Forsberg G, Nordle O, Pili R, Morris MJ (2014). Assessment of the bone scan index in a randomized placebo-controlled trial of tasquinimod in men with metastatic castration-resistant prostate cancer (mCRPC). Urol Oncol.

[14] Armstrong AJ, Garrett-Mayer E, de Wit R, Tannock I, Eisenberger M (2010). Prediction of survival following first-line chemotherapy in men with castration-resistant metastatic prostate cancer. Clin Cancer Res.

[15] Beer TM, Armstrong AJ, Rathkopf DE, Loriot Y, Sternberg CN, Higano CS, Iversen P, Bhattacharya S, Carles J, Chowdhury S, Davis ID, de Bono JS, Evans CP, Fizazi K, Joshua AM, Kim CS, Kimura G, Mainwaring P, Mansbach H, Miller K, Noonberg SB, Perabo F, Phung D, Saad F, Scher HI, Taplin ME, Venner PM, Tombal B, Investigators, Prevail (2014). Enzalutamide in metastatic prostate cancer before chemotherapy. N Engl J Med.

[16] Mitsui Y, Shiina H, Yamamoto Y, Haramoto M, Arichi N, Yasumoto H, Kitagaki H, Igawa M (2012). Prediction of survival benefit using an automated bone scan index in patients with castration-resistant prostate cancer. BJU Int.

[17] Takahashi Y, Yoshimura M, Suzuki K, Hashimoto T, Hirose H, Uchida K, Inoue S, Koizumi K, Tokuuye K (2012). Assessment of bone scans in advanced prostate carcinoma using fully automated and semi-automated bone scan index methods. Ann Nucl Med.

[18] Cook GJ, Venkitaraman R, Sohaib AS, Lewington VJ, Chua SC, Huddart RA, Parker CC, Dearnaley DD, Horwich A (2011). The diagnostic utility of the flare phenomenon on bone scintigraphy in staging prostate cancer. Eur J Nucl Med Mol Imaging.

[19] Morris MJ, Molina A, Small EJ, de Bono JS, Logothetis CJ, Fizazi K, de Souza P, Kantoff PW, Higano CS, Li J, Kheoh T, Larson SM, Matheny SL, Naini V, Burzykowski T, Griffin TW, Scher HI, Ryan CJ (2015). Radiographic progression-free survival as a response biomarker in metastatic castration-resistant prostate cancer: COU-AA-302 results. J Clin Oncol.

[20] Scher HI, Halabi S, Tannock I, Morris M, Sternberg CN, Carducci MA, Eisenberger MA, Higano C, Bubley GJ, Dreicer R, Petrylak D, Kantoff P, Basch E, Kelly WK, Figg WD, Small EJ, Beer TM, Wilding G, Martin A, Hussain M, Prostate Cancer Clinical Trials Working, Group (2008). Design and end points of clinical trials for patients with progressive prostate cancer and castrate levels of testosterone: recommendations of the Prostate Cancer Clinical Trials Working Group. J Clin Oncol.

